# MicroRNA-146a is induced by inflammatory stimuli in airway epithelial cells and augments the anti-inflammatory effects of glucocorticoids

**DOI:** 10.1371/journal.pone.0205434

**Published:** 2018-10-09

**Authors:** Kristin A. Lambert, Alanna N. Roff, Ronaldo P. Panganiban, Scott Douglas, Faoud T. Ishmael

**Affiliations:** 1 Department of Medicine, Division of Pulmonary, Allergy and Critical Care Medicine, The Pennsylvania State University Milton S. Hershey Medical Center, Hershey, Pennsylvania, United States of America; 2 Department of Biochemistry and Molecular Biology, Penn State College of Medicine, Hershey, Pennsylvania, United States of America; University of Torino, ITALY

## Abstract

**Background:**

MicroRNAs (miRNAs) are emerging as central regulators of inflammation, but their role in asthma and airway epithelial cells is not well studied. Glucocorticoids are the cornerstone of therapy in asthma and other inflammatory disease, yet their mechanisms of action are not completely elucidated, and it is not clear whether miRNAs modulate their effects.

**Objective:**

We aimed to identify miRNAs that regulate cytokine and chemokine expression in airway epithelial cells and whether these miRNAs are subject to the effects of glucocorticoids.

**Methods and results:**

MicroRNAomic analyses of immortalized, normal human bronchial epithelial cells identified 7 miRNAs that were altered by inflammatory cytokine treatment and 22 that were regulated by glucocorticoids (n = 3 for each treatment condition). MiR-146a emerged as a central candidate, whose expression was induced by TNF-α and repressed by glucocorticoids. Its role as a candidate in asthmatic inflammation was supported by expression profiling in human asthmatics, which showed that plasma miR-146a expression was elevated in asthma and associated with measures related to worse asthma outcomes, including elevated blood eosinophil counts, higher asthma control questionnaire scores, and need for higher doses of inhaled glucocorticoids. However, transfection of miR-146a in A549 cells treated with TNF-α +/- glucocorticoids produced an anti-inflammatory effect and increased efficacy of glucocorticoids.

**Conclusions:**

We propose a model whereby miR-146a is induced by inflammatory conditions as a feedback mechanism to limit inflammation. Exogenous administration of miR-146a augmented the effects of glucocorticoids and could be a novel therapeutic strategy to enhance efficacy of these medications.

## Introduction

The airway epithelium is an immunologically active barrier in the lung, producing cytokines and other inflammatory and chemotactic mediators in response to antigen, pollutant and allergen exposure from the external environment [1]. The epithelium interface senses allergic or microbial insult via innate pattern recognition receptors and responds by recruiting and activating leukocytes to sustain a cycle of chronic pulmonary inflammation central to asthma pathogenesis [2]. As the key location for initiation, exacerbation, and persistence of asthmatic inflammation, the airway epithelium is also the principal target for anti-inflammatory therapies like inhaled corticosteroids (ICS) and a key site for investigating the regulation of inflammation in asthma.

Our research has aimed to understand how de-regulation of inflammatory mediators in airway epithelial cells contributes to asthma, a chronic disease of the lungs, characterized by airway inflammation, reversible airflow obstruction, excess mucus production, bronchial hyper-responsiveness and airway remodeling [3]. Glucocorticoids (GCs), a mainstay in current asthma therapy, target and treat the inflammatory component of asthma [4]. Although the majority of asthmatic patients respond well to ICS, between a quarter and a third of individuals with poorly controlled asthma do not show improvement in pulmonary function despite treatment with high-dose, inhaled, or in some cases, oral corticosteroids [5, 6]. This subset of ‘glucocorticoid-resistant’ or ‘steroid-insensitive’ patients often have the worst disease control, are at greatest risk for asthma morbidity and mortality, and contribute disproportionately to healthcare expenditure [7, 8]. A lack of alternative treatments for severe asthmatic and steroid-insensitive patients supports investigations into therapeutics that may improve the efficacy of existing ICS therapy. The identification of such therapeutics relies on an evolving understanding of molecular mechanisms that regulate inflammation and glucocorticoid effects in the airway.

Post-transcriptional regulation (PTR) is an essential control mechanism for the expression of genes involved in inflammation like cytokines and chemokines [9, 10]. We previously found that a majority of epithelial-derived cytokines that are susceptible to post-transcriptional regulation are not Th2-driven, but rather highly induced by tumor necrosis factor alpha (TNF-α), a general inflammatory stimulus and pathogenic cytokine in severe, corticosteroid-refractory asthma [9, 11–14]. Severe asthma in human subjects and in animal models has been correlated with increased levels of TNF-α in bronchoalveolar lavage and lung biopsies [15]. Importantly, *in vitro* and *in vivo* investigations have also demonstrated that airway inflammation and hyper-responsiveness in asthma are dampened by TNF-α deficiency or antagonism with anti-TNF-α monoclonal antibodies[16, 17]. An important inducer of inflammation that also contributes to glucocorticoid resistance, TNF-α’s effects may be subject to PTR by microRNAs (miRNAs) in airway epithelium.

Small functional non-coding RNAs, miRNAs, participate in post-transcriptional regulation of inflammatory genes by binding to complementary target mRNAs and inhibiting their translation. Previous work by our group and others has demonstrated that miRNAs have central roles in homeostatic immune regulation and pathogenic disease when dysregulated. We have previously shown that miRNAs are differentially expressed in asthmatics compared to healthy, non-asthmatic controls and are critical regulators of allergic inflammation [18, 19]. Recent studies have also identified that glucocorticoids can alter expression of miRNAs involved in proinflammatory responses [20]; however, little is mechanistically known about the role of miRNAs in mediating the suppressive effects of glucocorticoids in inflammation.

In this study we hypothesized that miRNAs regulate inflammatory mediators in airway epithelial cells and that glucocorticoids modulate miRNAs as part of its anti-inflammatory mechanism of action. We identified miRNAs that are altered by inflammatory stimuli and glucocorticoids in the airway epithelium. In particular, we demonstrate that miRNA-146a is an anti-inflammatory molecule, which augments the effects of glucocorticoids. These findings are discussed herein.

## Materials and methods

### Patient selection

This study was approved by the Penn State College of Medicine Institutional Review Board. In accordance with approved protocols, written informed consent was obtained from all patients. Study participants were included based on established clinical history of asthmatic symptoms and objective measures of pulmonary function at the time of study enrollment (Table 1). Asthmatic subjects were identified based on forced expiratory volume in one second (FEV_1_) reversal ≥12% and ≥200 ml post-bronchodilator, or airway hyper-responsiveness induced by methacholine (provocating concentration producing a 20% fall in FEV_1_ of less than 8 mg/mL). All asthmatic patients completed the seven question asthma control questionnaire (ACQ-7) to assess asthma control [21]. Blood was collected from subjects by venipuncture and total RNA was extracted from serum, processed, and analyzed for qPCR analysis as previously described [19].

**Table 1:** Human subject characteristics.

Characteristics	Healthy Subjects (n = 19), no. (%) or mean ± SEM	Asthmatic Subjects (n = 35), no. (%) or mean ± SEM	P value
Age (y)	41.5 ± 3.25	43.9 ± 2.47	0.563 *
**Sex**			
Male/female, no./no. (%/%)	8/11 (42/58)	14/21 (40/60)	0.957 †
**Ethnicity**			
White/nonwhite, no./no. (%/%)	16/3 (85/15)	34/1 (97/3)	0.129 †
BMI (kg/m^2^)	27.7 ± 1.64	29.2 ± 1.09	0.701 *
Smoker, no. (%)	5 (26)	5 (14)	0.268 †
**Spirometry**			
FVC (L)	3.94 ± 0.34	3.70 ± 0.19	.126 *
FVC (% predicted)	93.3 ± 4.55	89.9 ± 2.84	0.081 *
FEV_1_ (L)	3.05 ± 0.29	2.68 ± 0.18	0.036 *
FEV_1_ (% predicted)	87.3 ± 4.94	78.4 ± 3.85	0.008 *
FEV_1_/FVC ratio	0.76 ± 0.02	0.70 ± 0.02	0.041 *

* Normally distributed continuous variables were analyzed by using one-way ANOVA.

† Freeman-Halton extension of the Fisher exact test was used to analyze categorical binary data.

### Tissue culture

A549 human lung carcinoma cells (ATCC) were cultured in Eagle’s F12K media (ATCC) supplemented with 10% FBS, 100 U/mL penicillin, and 100 μg/ml streptomycin (Invitrogen). NuLi-1 immortalized, normal human bronchial epithelial cells (ATCC) were grown in serum-free BEGM (Lonza) without gentamycin-amphotericin B and supplemented with 50 μg/ml G418 (Invitrogen). All cells were maintained in a 37°C incubator with 5% CO_2_.

### Transient transfections

For overexpression of miR-146a, A549 cells were grown to 70–90% confluence in tissue-culture-treated, 6-well plates and transfected with 10 nM AllStars Negative Control siRNA (Qiagen) or miR-146a-5p mimic (Qiagen) using RNAiMAX (Invitrogen) as described by the manufacturer. Media was replaced 6 hours post-transfection with fresh medium using charcoal-stripped FBS and then treated with vehicle, 50 ng/ml TNFα (PeproTech), and/or 10 μM Dexamethasone (Sigma-Aldrich) as indicated. Cells and media were harvested for isolation of both RNA and protein 18 hours later (24 hour post-transfection).

### RNA isolation, cDNA synthesis, and quantitative real-time PCR

Total RNA was isolated by TRIzol reagent (Invitrogen) and the Driect-zol RNA Miniprep Kit (Zymo Research) as described by the manufacturers and quantified by UV absorbance at 260 nm. cDNA synthesis for mRNA was done using 2μg RNA and the High Capacity cDNA Reverse Transcription Kit (Applied Biosystems) according to the manufacturer’s instructions, and for miRNA using 1 μg RNA and the qScript microRNA Reverse Transcription Kit (Quanta Biosciences). Quantitative Real-Time PCR was performed using template cDNA, 2x iTAq Universal SYBR Green SuperMix (BioRad), and the gene-specific primers. All reactions were run in 96-well plates on the MyiQ2 Two Color Real-Time Detection System (BioRad) using the following program: 2 min hot start at 95° C, 40 cycles of 15 s at 95° C and 30 or 60 s at 60° (for miRNA and mRNA, respectively), and 2 min at 10° C. Data were analyzed by the ΔΔCt method with normalization to the endogenous control GAPDH (mRNA) or snoRD44 (miRNA).

### MicroRNA microarray analysis

Microarray analysis of miRNA expression in NuLi cells treated with TNF-α, dex, TNF-α+dex, or neither (n = 3 for each condition) was performed by Ocean Ridge Biosciences. A full description of the methodology is available at the company’s website: (https://www.oceanridgebio.com/protocols_and_methods). RNA integrity was quality controlled using gel electrophoresis and analyzed using the company’s miRBase v19 miRNA microarray, which contains probes for 2,040 human miRNAs. Statistical significance was calculated using the False Discovery Rate (FDR) method which was proposed by Benjamini and Hochberg, using a threshold of 0.2. We selected miRNAs for further study and validation by qPCR if the FDR was ≤0.2 or P≤0.05. Principal component analysis (PCA) of each condition, TNF-α, dex, and TNF-α + dex is provided in supplemental information (S1 Fig).

### ELISA, Luminex assays

Secreted CCL2 protein was quantified from A549 cell culture media using the Quantikine ELISA Human CCL2/MCP-1 Immunoassay (R&D Systems) as described by the manufacturer. Samples were diluted 1:10 in the appropriate Calibrator Diluent, absorbances measured on a GENios plate reader (Tecan) and 4-PL standard curves generated using Assay Blaster! Data Analysis Software (Enzo Life Sciences). Secreted CCL5/RANTES, IL-6, GM-CSF,and IL-8 proteins were also quantified from A549 cell culture media using a custom Lumniex Performance Assay (R&D Systems) as described by the manufacturer. Undiluted, 1:10 and 1:20 dilutions of sample were run on a BioPlex 200 instrument (Bio-Rad) and 5-PL standard curves were generated using the included software. Samples that were outside the lower limit for detection were considered for statistical purposes to have the minimum detectable dose (MDD) as defined by the manufacturer for each individual protein.

### Statistical analysis

Differences in miRNA expression between asthmatic and non-asthmatic subjects was determined by Mann-Whitney U test, with a significance set at p<0.05. Association between eosinophil count and miRNA expression, asthma control questionnaire (ACQ) score and miRNA expression, and ICS dose and miRNA expression were determined by Pearson correlation testing, with a significance set at p<0.05. Hypothesis testing was performed by one-way ANOVA with Bonferroni-Holm post-hoc tests using Prism (GraphPad Software, Version 4.0).

## Results

### Identification of miRNAs regulated by inflammatory stimuli or glucocorticoids

**Fig 1:**
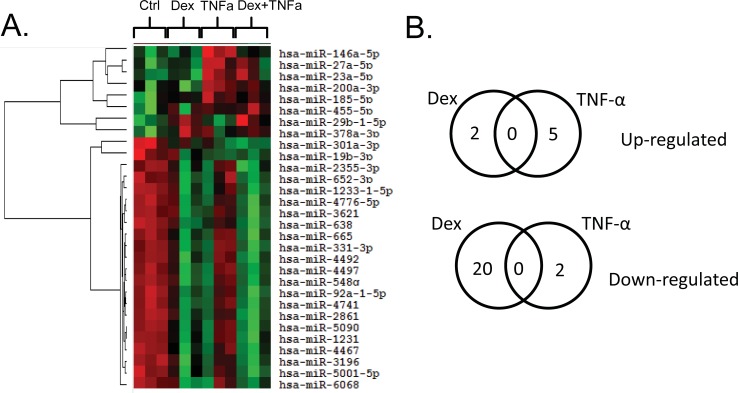
MicroRNAomic analyses of primary bronchial epithelial cells identify miRNA candidates central to inflammatory processes and altered by steroids in the airway. A) Heatmap of miRNA expression in NuLi cells treated with or without TNF-α ± dexamethasone. Each treatment condition in the heatmap is the result of an n of 3. Green is lower expression and red is higher expression in the heat map. B) Venn diagram showing miRNAs upregulated and downregulated with respective treatments.

**Fig 2:**
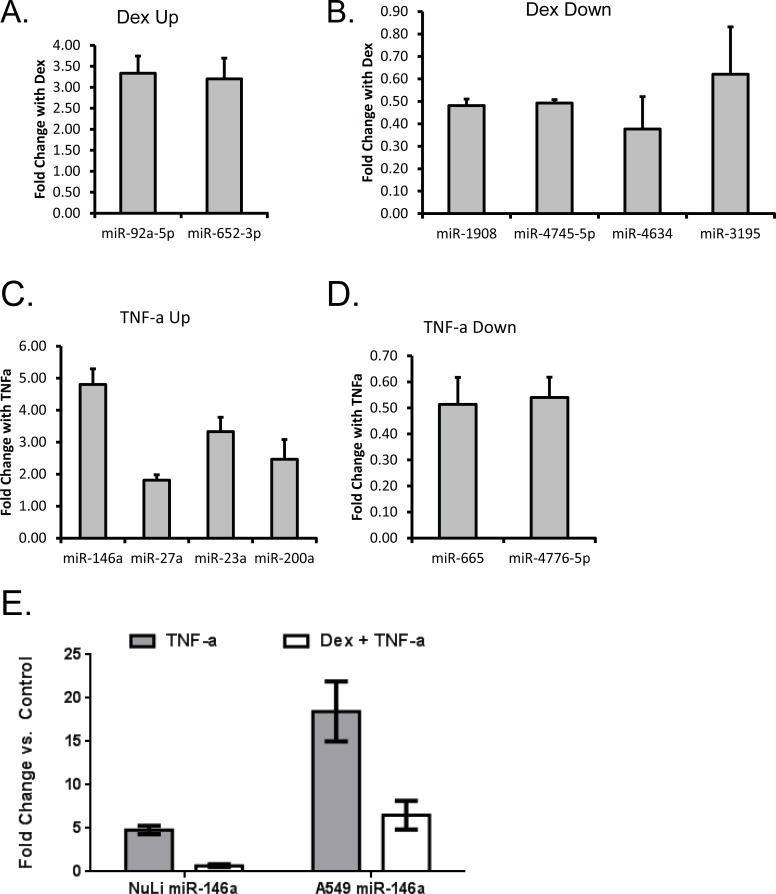
MiRNA candidates altered by TNF-α, but antagonized or augmented by dexamethasone. NuLi cells were treated with dexamethasone (A and B), TNF-α (C and D) and miRNA candidates identified by microarray were validated by qRT-PCR. E) NuLi cells and A549 airway epithelial cells were treated with TNF-α alone or TNF-α + dexamethasone and miRNA-146a was measured by qRT-PCR. miRNA levels were normalized to snord44, and expression was calculated as fold change relative to no treatment control. Results are shown as the mean ± SEM, n = 3. * p<0.05.

We first sought to identify airway epithelial cell miRNAs that were altered by TNF- α and whether these effects were antagonized by glucocorticoids (GCs). Immortalized normal human bronchial epithelial cells (NuLi-1) were treated with/without TNF-α (50 ng/ml) and/or dexamethasone (dex, 1 μM) for 24 hours, followed by isolation of RNA and analysis using miRNA microarray. There were 660 miRNAs that were detectable in the airway epithelial cells in the absence of treatment (S1 File). Using a criteria of FDR ≤0.2 or P≤0.05, we identified miRNAs for further study: five miRNAs were up-regulated by TNF- α and two down-regulated, and two miRNAs were up-regulated by GCs and 20 downregulated (Fig 1A, 1B, and Table 2). None of the dex up-regulated miRNAs were in common with TNF-α up-regulated miRNAs, and this held true for miRNAs down-regulated by each stimuli. We next asked whether there were any miRNAs that were induced by TNF-α and repressed by GCs, as these could constitute pro-inflammatory miRNAs that could be targeted by GCs. Comparing the effects of TNF-α alone vs. TNF-α + dex, miR-146a was the only miRNA that showed statistical differences. The top candidates identified as up- or down-regulated by the stimuli in the microarray experiment were validated by quantitative real time PCR (qPCR) (Fig 2A–2E). The qPCR results were consistent for all of the miRNAs tested except for miR-3195 (Fig 2B) and miR-200a (Fig 2C). The up-regulation of miR-146a by TNF-α and its inhibition by GCs were also confirmed by qPCR in NuLi cells, an immortalized, normal human bronchial epithelial cell line that is widely used to study normal human airway epithelial cell function (Fig 2E). We observed a 4.80 ± 0.49 fold change in miR-146a following TNF-α stimulation in NuLi cells and this change was abolished by glucocorticoids. Dexamethasone antagonism of TNF-α caused a 0.68 ± 0.16 fold change in miR-146a in NuLis. Importantly, the same trends were observed in a second airway epithelial cell line, A549s, where miR-146a levels increased in response to TNF-α stimulation and were attenuated with the addition of dex. Unlike TNF-α, traditional Th2 stimuli did not cause a marked change in miR-146a expression in either NuLi or A549 cells (S2 Fig). We selected A549 cells for the complementary analysis, as these cells are easily transfectable with miRNAs, in contrast to the NuLi cells.

**Table 2:** MiRNAs altered by TNF- α or dex.

miRNA	p-value	Fold change	FDR value (aka q-Score)
**Up-regulated by TNF-α**			
hsa-miR-146a-5p	0.006	11.56	0.2264
hsa-miR-27a-5p	0.012	3.60	0.275308
hsa-miR-185-5p	0.045	3.03	0.417336
hsa-miR-200a-3p	0.007	2.64	0.2264
hsa-miR-23a-5p	0.004	2.47	0.2264
**Down-regulated by TNF-α**			
hsa-miR-665	0.003	0.48	0.2264
hsa-miR-4776-5p	0.013	0.47	0.275308
**Up-regulated by Dex**			
hsa-miR-92a-1-5p	0.001	2.11	0.068787
hsa-miR-652-3p	0.047	2.00	0.22756
**Down-regulated by Dex**			
hsa-miR-4497	0.015	0.50	0.124161
hsa-miR-3621	0.006	0.50	0.088041
hsa-miR-638	0.003	0.50	0.082722
hsa-miR-1233-1-5p	0.010	0.49	0.100737
hsa-miR-2861	0.003	0.49	0.076561
hsa-miR-548q	0.014	0.49	0.121965
hsa-miR-4741	0.008	0.48	0.089151
hsa-miR-5090	0.007	0.48	0.088041
hsa-miR-4492	0.001	0.48	0.068787
hsa-miR-1231	0.014	0.47	0.121965
hsa-miR-3196	0.004	0.47	0.082722
hsa-miR-5001-5p	0.012	0.45	0.111989
hsa-miR-6068	0.003	0.43	0.076855
hsa-miR-4467	0.006	0.43	0.088041
hsa-miR-3178	0.007	0.43	0.088041
hsa-miR-1469	0.010	0.42	0.100737
hsa-miR-4745-5p	0.010	0.42	0.100737
hsa-miR-4634	0.005	0.41	0.088041
hsa-miR-3195	0.028	0.39	0.162879
hsa-miR-1908	0.006	0.36	0.088041
Up-regulated by TNF-α, inhibited by Dex			
hsa-miR-146a-5p	0.00591 ^a^	0.16^b^	0.918264

^a^ p-value Dex+TNF-α vs TNF- α

^b^ Fold change Dex+TNF-α vs TNF- α

### Analysis of candidate miRNA function

In order to begin to understand the potential function of candidate miRNAs involved in inflammatory or glucocorticoid pathways, we performed in silico analyses. Using DIANA-miRPath v.3, we examined the KEGG pathways regulated by miRNAs altered by TNF-α or dex treatment (Table 3). Based on the number of genes regulated by miRNAs, the top KEGG pathways identified were “proteoglycans in cancer”, “toll-like receptor (TLR) signaling pathway”, “NF-kappa-B signaling pathway”, “ECM receptor interaction”, “glycosaminoglycan biosynthesis”, and “biosynthesis of unsaturated fatty acids”. Many of these pathways represent key defense mechanisms for inflammation, infection and innate immunity in the lung and are, in part, regulated by our top candidate, miR-146a.

**Table 3:** Pathway analysis.

KEGG Pathway	Genes Targeted	miRNAs that target
Biosynthesis of unsaturated fatty acids	ACOT1, ACOT2, ACOT4, SCD5	miR-665, miR-200a-3p
Toll-like receptor signaling pathway	MYD88,TIRAP,IRF5,CXCL10, TRAF6, IRAK1, TLR4, IFNAR2, MAP2K4, RIPK1, RELA, PIK3R2	miR-665, miR-146a, miR-23a-5p, miR-200a, miR-4776-5p, miR-185-5p
NF-kappa B signaling pathway	RELA, CXCL12, MYD88,TIRAP, PIDD1, TRAF6, IRAK1, RIPK1, TLR4	miR-665, miR-146a, miR-23a-5p, miR-200a, miR-4776-5p, miR-185-5p
Glycosaminoglycan biosynthesis	HS6ST2, EXT1, HS3ST2, B3GAT3	miR-665, miR-200a-3p, miR-27a-5p
Proteoglycans in cancer	SDC1,THBS1,CAV1,WNT4 TLR4,PPP1R12B,HPSE,DCN CTNNB1,IGF2,TIMP3,TGFB2 KDR,CD44,PRKACB,PIK3R2	miR-665, miR-146a, miR-23a-5p, miR-200a, miR-4776-5p, miR-185-5p
ECM-receptor interaction	GP5,SDC1,THBS1,COL24A1 COL6A6,ITGA6,CD44,COL4A1	miR-665, miR-27a-5p, miR-200a, miR-4776-5p,

### miR-146a has an anti-inflammatory effect in airway epithelial cells

**Fig 3:**
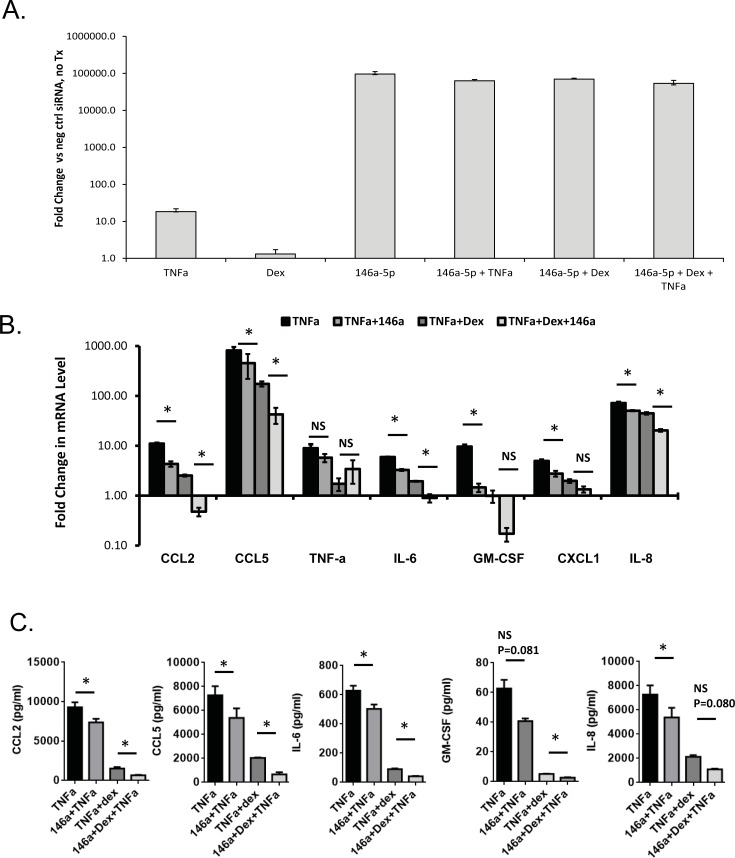
Effect of miR-146a and dexamethasone on repression of TNF-α induced cytokines in airway epithelial cells. A) A549s were transfected with miR-146a mimic or negative control small RNA, treated with vehicle or TNF-α ± dexamethasone. Confirmation of successful transfection of miR-146a is shown relative to negative control (scrambled siRNA). B). RNA was extracted and analyzed by qRT-PCR. Epithelial cytokine gene levels were normalized to GAPDH, and expression was calculated as fold change relative to the negative control miRNA transfection without TNF-α stimulation and dexamethasone treatment. Results are shown as mean ± SEM, n = 3. * p<0.05. C) Secreted proteins were measured in A549 cell culture medium by ELISA. Results are shown as the mean ± SEM, n = 3. * p<0.05.

After showing that miR-146a is induced by inflammatory stimuli and repressed by steroids in two independent cell systems, we sought to further investigate the immunoregulatory function of our top target, miR-146a, in the A549 epithelial line because it is easily transfected, in contrast to normal human bronchial epithelial cells. To determine the contributions of miR-146a to post-transcriptional regulation of cytokines in airway epithelial cells, A549s were transiently transfected with a miR-146a mimic or negative control small RNA, and 6 h later treated with TNF-α +/- dex for 18 h (i.e. total transfection time of 24 h). Successful transfection of miR-146a-5p mimic was confirmed in untreated and treated A549s (Fig 3A). Gene expression changes in a panel of epithelial cytokines were measured by qPCR and shown as fold change relative to negative miRNA control without any stimulation. As expected, TNF-α treatment induced robust gene expression and cytokine protein changes in the panel we studied (Fig 3B and 3C). Overexpression of miR-146a attenuated the TNF-α-induced expression of most of the cytokines, with statistically significant gene repression (determined by ANOVA with Tukey correction for multiple comparisons, significance threshold of P<0.05) of CCL2, CCL5, IL-6, GM-CSF, CXCL1, and IL-8 (Fig 3B). The level of miR-146a-induced repression was not as large as the effects of dex, a well-established anti-inflammatory glucocorticoid. However, the combination of miR-146a and dex provided better inhibition of cytokine production than dex alone, and this trend was observed for the majority of cytokines (statistically significant effects observed for CCL2, CCL5, IL-6, IL-8, comparing effects of TNF-α+dex vs. TNF-α+dex+miR-146a). To confirm that miR-146a also produced effects on cytokine protein expression, a Luminex assay was used to measure expression in a representative panel of cytokines (CCL2, CCL5, IL-6, IL-8, GM-CSF). The effects of miR-146a were consistent, though effects of miR-146a+TNF-α vs. TNF-α on GM-CSF (p = 0.081), and effects of TNF-α +dex vs. TNF- α +dex+miR-146a (p = 0.080) did not reach statistical significance (Fig 3C).

### miR-146a enhances the efficacy of dexamethasone, but does not alter potency

**Fig 4:**
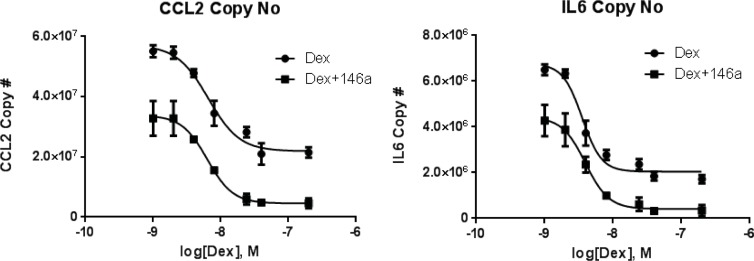
Concentration dependent effects of miR-146a on cytokine repression in airway epithelial cells. A549s were transfected with miR-146a mimic or negative control small RNA, stimulated with TNF-α, and then dexamethasone was added at increasing doses. CCL2 and IL-6 levels were measured by qRT-PCR. Results are shown as the mean ± SEM, n = 3.

To further characterize the anti-inflammatory effects of miR-146a, we performed a dose response experiment where cells were transfected with miR-146a or negative control, stimulated with TNF-α, and dex was added at increasing concentrations. Effects on CCL2 and IL-6 expression were measured as representative cytokines that we previously demonstrated to be important airway epithelial GC targets [9, 11, 12]. Consistent with the data presented in Fig 3, miR-146a transfection repressed the TNF-α-induced expression of CCL2 and IL-6 at all concentrations of dex used in this study compared to dex alone (negative control miRNA transfection + dex) (Fig 4).The IC_50_ values for dex inhibition of either cytokine did not change with miR-146a (Fig 4). For IL-6, the IC_50_ for dex was 3.54 x10^-9^ M in the absence of miR-146a and 3.94x10^-9^ M in the presence of miR-146a, and the IC_50_ values for CCL2 were 6.52x10^9^ M (- miR-146a) and 6.33x10^9^ M (+ miR-146a). However, the maximal effect of dex on repression of each cytokine was increased by miR-146a. Maximal effect of dex on repression of cytokine expression (copies/1000 cells) + miR-146a vs.–miR-146a was 4.55x10^6^ vs. 2.20x10^7^ for CCL2 and 4.11x10^5^ vs. 2.04x10^6^ for IL-6 (Fig 4).

### Circulating plasma miR-146a expression is elevated in human asthmatics and correlates with measures of poor disease control

**Fig 5:**
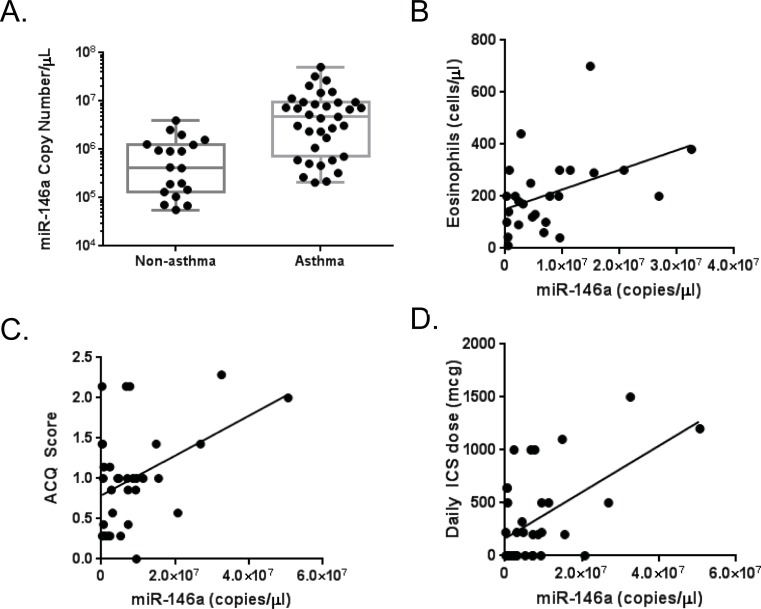
Expression of miR-146a in blood of asthmatics vs. non-asthmatics and its association with eosinophil levels, ACQ score, and ICS dose. A) Plasma concentration of miR-146a was measured in asthmatic (n = 35) and non-asthmatic healthy controls (n = 19) using qRT-PCR. Asthmatic individuals had a median of 4.73x10^6^ copies of miR-146a/μL vs. healthy individuals who had a median of 4.24x10^5^ copies/μL. Mann Whitney test determined significance at p < 0.05. B) Eosinophil counts, C) Asthma Control Questionnaire (ACQ) score, and D) daily inhaled corticosteroid (ICS) dose correlated with plasma miR-146a copy number in asthmatic subjects (n = 35). R = correlation coefficient. * p<0.05.

The induction of miR-146a by inflammatory stimuli in airway epithelial cells *in vitro* implicated it as a potentially important miRNA, and raised the question of whether it could play roles in inflammatory airway disease. We previously demonstrated that blood miRNA expression profiles were different in asthmatic vs. non-asthmatics, and that these differentially expressed miRNAs had potential regulatory roles in inflammation. To determine whether miR-146a had any potential roles in human asthma, we analyzed its expression and relationship with clinical features in a data set of asthmatic (n = 35) and non-asthmatic (n = 19) that we previously characterized [19]. Circulating plasma concentrations of miR-146a were significantly elevated in asthmatic individuals compared to healthy controls (Fig 5A), consistent with our *in vitro* observations that miR-146a was up-regulated under inflammatory conditions in airway epithelial cells. We also found that expression of plasma miR-146a was positively associated with blood eosinophil levels in asthmatics, which can be a marker of worse asthma outcomes (Fig 5B) [22]. Accordingly, miR-146a levels were also associated with higher asthma control questionnaire (ACQ-7) scores, indicating that levels were higher in asthmatics with worse disease control (Fig 5C). Furthermore elevated blood miR-146a levels in asthmatics were also associated with need for higher doses of ICS, which may be a marker for asthma that is harder to treat (Fig 5D).

## Discussion

miRNAs contribute to normal cellular processes in both innate and adaptive arms of immunity, controlling development, maturation, differentiation and activation of immune cells [23–25]. Dysregulation of miRNAs is known to contribute to the pathogenesis of various inflammatory diseases, including rheumatoid arthritis [26, 27] and inflammatory bowel disease [28], though their mechanisms in asthma are not well defined. Increasing evidence implicates airway epithelium as a critical contributor to immune responses in the lungs, and, in parallel, raises the possibility that miRNAs play important roles in these cells. In this study we aimed to identify miRNAs in airway epithelial cells that are altered by inflammation and glucocorticoids, determine whether these may be deregulated in asthma, and whether they may be targets for anti-inflammatory approaches.

A known player in asthma pathogenesis, TNF-α was used as a stimulus in this study to better model non-Th2 forms of asthma that are often associated with severe outcomes and glucocorticoid insensitivity. We demonstrated in two airway epithelial cell lines that traditional Th2 stimuli have negligible effects on miRNA expression in airway cells compared to TNF-α, making our non-Th2 stimulated system better for interrogating miRNA-mediated changes in the airway epithelium. Using this model, we identified numerous miRNAs whose expression in airway epithelial cells was altered by TNF-α, GCs, or both. Pathway analyses indicated that these miRNAs may have potential roles in inflammation, and targets were mapped to TLR-signaling and the NFκB pathway. These findings suggest a role of airway epithelial cell miRNAs in innate immunity and infection, consistent with their important site at the interface between host and environment. Furthermore, our findings that miRNAs may participate in extracellular matrix-receptor interactions are consistent with our previous work indicating that miRNAs may regulate processes involved in asthmatic remodeling of airways[29]. We note that miR-146a makes specific contributions to regulating members of all of these pathways that are crucial to airway epithelial inflammatory processes and asthmatic inflammation.

miR-146a has been previously reported to be an important molecular brake or suppressor of inflammation through its capacity to target members of TLR signaling, NFκB signaling and the proteoglycan family. Initial studies by Taganov et al., conducted in TNF-α stimulated monocytes, were the first to demonstrate a functional role of miR-146a in regulating NFκB activation via toll-like receptor signaling [30]. Common to both NFκB and TLR pathways, genes IRAK1 and TRAF6 are directly targeted and downregulated by miR-146a [30–33]. We identified IRAK1 and TRAF6 as pathway genes susceptible to miR-146a regulation in airway epithelial cells. Studies in cancer biology have also identified miR-146a as a regulator of proteoglycan synthesis, which may be relevant to the control of mucoprotein deposition in asthma that contributes to airway remodeling.

Our work identified miR-146a as a potentially important candidate in regulation of airway inflammation. Previous reports have implicated miR-146a as an immunomodulatory miRNA, with the majority of studies reporting functions that inhibit inflammation [34, 35]. Consistent with these widespread observations, we demonstrated that miR-146 is induced by inflammatory stimuli and its overexpression in airway epithelial cells represses multiple proinflammatory cytokines. We assert that miR-146a is induced as a feedback regulator in order to limit the amplitude and timing of the inflammatory response in asthma. Along these lines, numerous studies have shown that miR-146a serves as a negative feedback regulator of NFκB signaling in human cells, innate immune signaling through toll-like receptors, and cytosolic receptor signaling for antiviral defense [30, 32, 36–42]. Most recently, miR-146a-5p was shown to suppress NFκB-inducible cytokine, CCL20, in airway smooth muscle cells of asthmatic and healthy individuals. This work implicates miR-146a as not only an important regulator of NFκB-dependent-inflammation but also asthma, specifically mucus secretion [43].

In accordance with our observations, it has been reported that miR-146a reduces NFκB-dependent proinflammatory cytokines in TNF-α stimulated monocytes [30]. Our results extend these findings to airway epithelial cells, indicating that miR-146a is not only expressed in these cells, but also altered by disease, drugs and inflammatory conditions. Outside of our study, only two others have provided evidence for miR-146a-mediated PTR in airway epithelial cells, Liu et al demonstrated that miR-146a inhibits NFκB signaling in airway epithelial cells in response to particulate-matter induced inflammation[44] and Huang et al noted that miR-146a is also mechanosensitive, participating in PTR in lung epithelial cells of small airways in response to mechanical force and pressure-induced inflammation[45].

Our observations that miR-146a was up-regulated in the blood of asthmatics and associated with markers of poor asthma control (e.g. ACQ score), also suggests that miR-146a is produced in response to damage in the airway and released into the circulation as a compensatory mechanism to attenuate chronic inflammation in human asthmatics. While the exact source of extracellular, plasma miR-146a has not yet been confirmed, we suspect that circulating leukocytes are the main contributor to high blood levels of miR-146a, since miR-146a expression is largely restricted to immune cells [46]. Immune cells like other cell types are capable of releasing RNA into the extracellular environment in forms that include, naked or unbound RNA, RNA-protein complexes and RNA enclosed in nanosized, lipid membrane extracellular vesicles. Increased secretion of miR-146a by leukocytes may be a basis for elevated plasma miR-146a.

However, it is important to point out that blood levels may not necessarily correlate with levels of the miRNA in the airway, and this will need further study. The elevation in the blood in asthmatics (and particularly those with worse symptom control) could purely be a marker for severity of inflammation. We will also need more in depth studies to determine whether the anti-inflammatory effects of miR-146a are preserved in asthma. It is possible that although the miRNA is able to be up-regulated, that other factors affect the function of the miRNA. We also cannot exclude the possibility that miR-146a could have pro-inflammatory effects in cells other than airway epithelium, such that elevation in the blood of asthmatics is the product of the effects of a different cell type.

Glucocorticoids have well-established suppressive effects on inflammation; however, their mechanisms of action have not been completely elucidated, particularly in airway epithelial cells. Moreover, resistance to glucocorticoids is emerging as an important barrier to effective treatment in severe asthma. Our findings raise the possibility that the mechanisms of GCs are intertwined with miRNA expression and function. It was interesting to note that GCs repressed miR-146a expression in primary human airway epithelial cells. This effect may result from the intersection of miR-146a as a feedback inhibitor of NFκB signaling and GCs as potent inhibitors of NFκB [47]. By inhibiting NFκB function, GCs may also remove natural feedback inhibitors of this pathway, like miR-146a. This line of reasoning would suggest that exogenous addition of miR-146a to GC-treated cells could enhance their effects. Indeed, we found that overexpression of miR-146a, when combined with dexamethasone, had greater anti-inflammatory effects than either by itself. This effect was due to an increase in efficacy of GCs, not potency. The exact mechanisms of action of miR-146a remain to be determined, but we hypothesize that the miRNA and GCs target distinct, though possibly related pathways. For instance, miR-146 has been shown to repress targets of NFκB signaling like IRAK1 and TRAF6 [30, 33, 41, 48, 49], while GCs are known act by trans-repression of the transcription factor [47].

The implication of these findings is that miR-146a replacement therapy could be approached as a novel strategy for boosting the anti-inflammatory effects of GCs in steroid-insensitive asthmatics. In the field of cancer biology, combination therapy is the gold standard of treatment and ongoing studies have pioneered miRNA replacement therapy in conjunction with chemotherapeutic agents [50]. Intranasal delivery of anti-inflammatory miRNAs has been shown to reduce airway inflammation in mouse models [51–53], indicating that these molecules have potential therapeutic benefit and could be introduced as an aerosol.

## Conclusions

Our findings suggest that airway epithelial cell miR-146a is an anti-inflammatory feedback inhibitor that enhances the effects of GCs, and that blood levels may be a marker of asthma. Further investigation into its mechanisms of action may lead to better biomarkers that predict glucocorticoid responsiveness and may be tapped therapeutically to overcome glucocorticoid resistance in severe asthmatic patients.

## Supporting information

S1 Fig Principal component analysis of treatment conditions or groups in microarray. (PDF)

S2 Fig Th2 vs. TNF-α induced miR-146a expression in airway epithelial cell lines. (PDF)

S3 Fig Baseline expression of cytokines for miR-146a or negative miRNA control transfection. (PDF)

S1 File Detectable miRNAs in microarray of TNF-α ± dex treated airway epithelial cells. (XLSX)
