# Supplementary figures and images for: MicroRNA-146a is induced by inflammatory stimuli in airway epithelial cells and augments the anti-inflammatory effects of glucocorticoids
Source: PLoS One. 2018 Oct 9;13(10):e0205434. doi: 10.1371/journal.pone.0205434 (PMC6177187; doi:10.1371/journal.pone.0205434)

Scores Plot

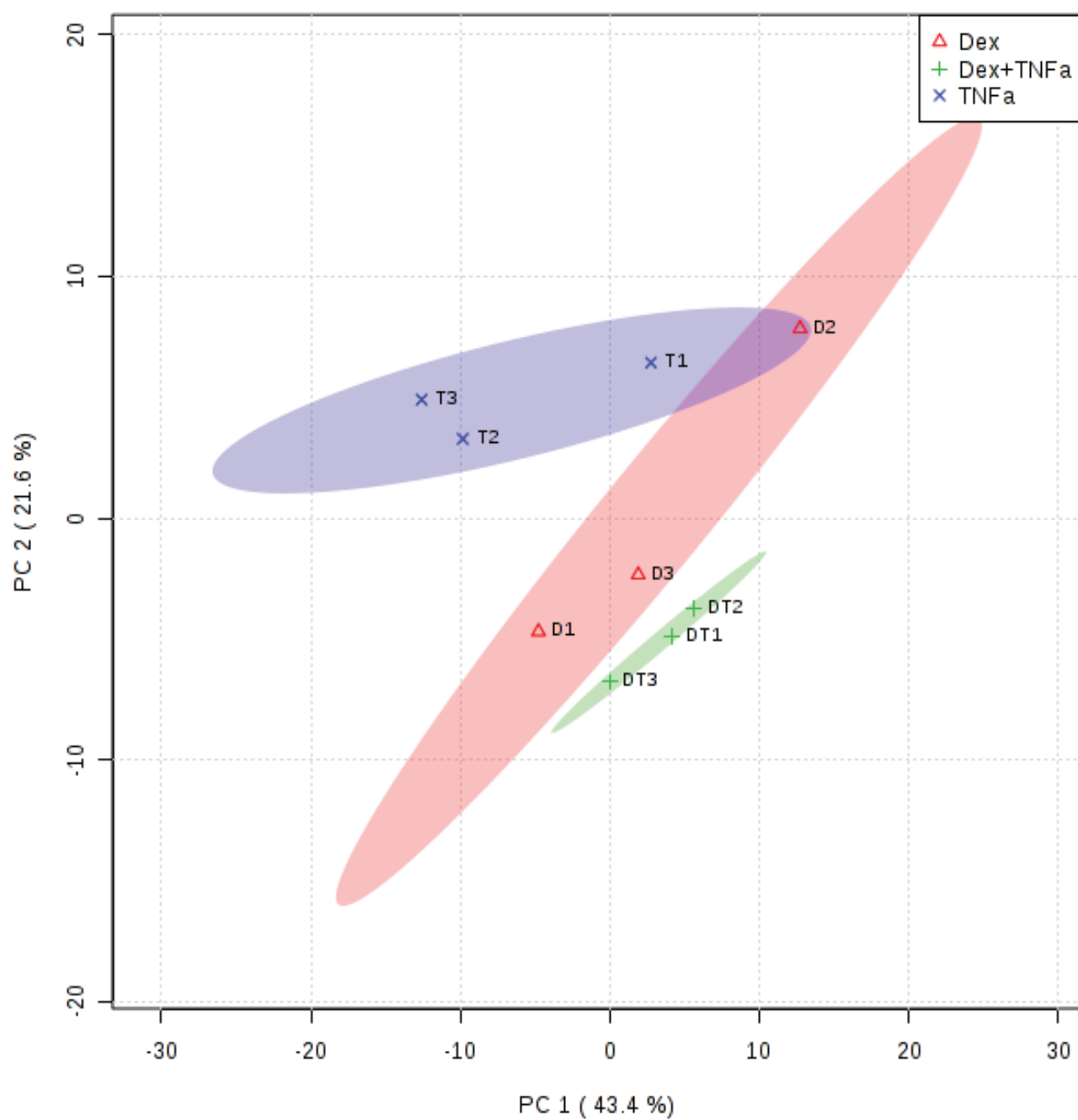

Supplement: S1 Fig — (PDF) [file pone.0205434.s001.pdf]

**miR-146a expression in airway epithelial cell lines**  
*Th2 vs. non-Th2 stimulus*

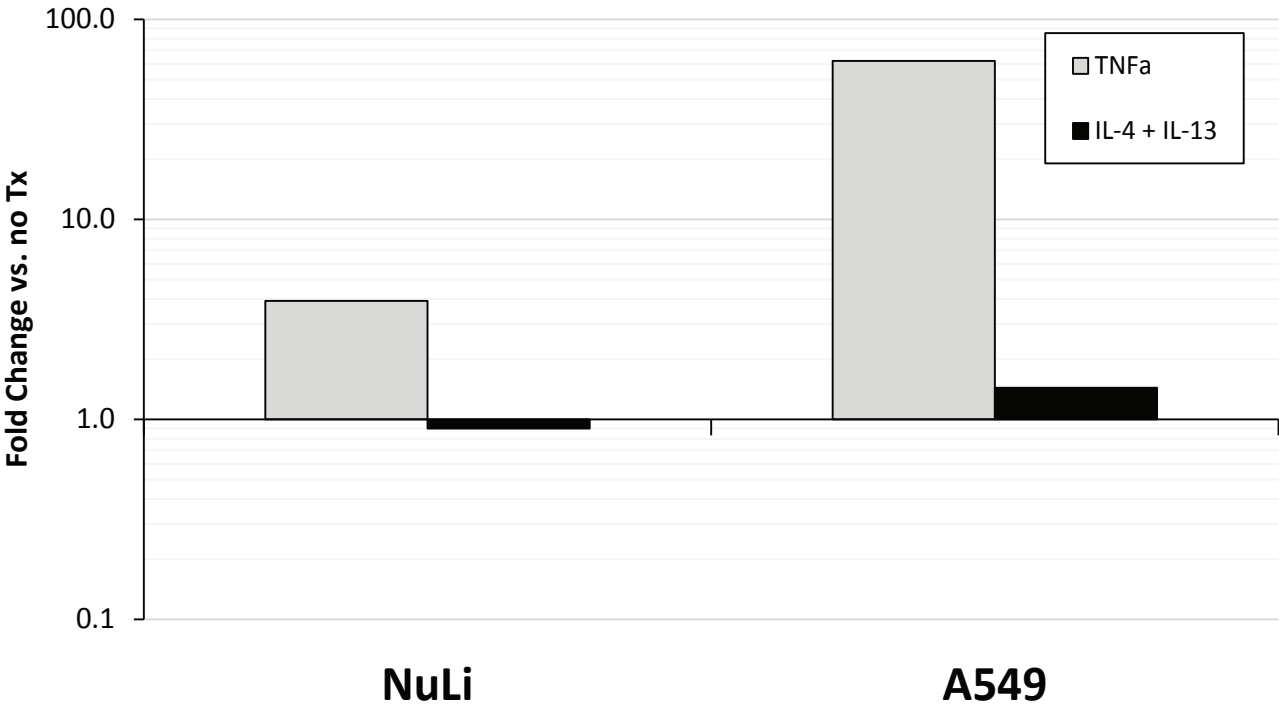

Supplement: S2 Fig — (PDF) [file pone.0205434.s002.pdf]

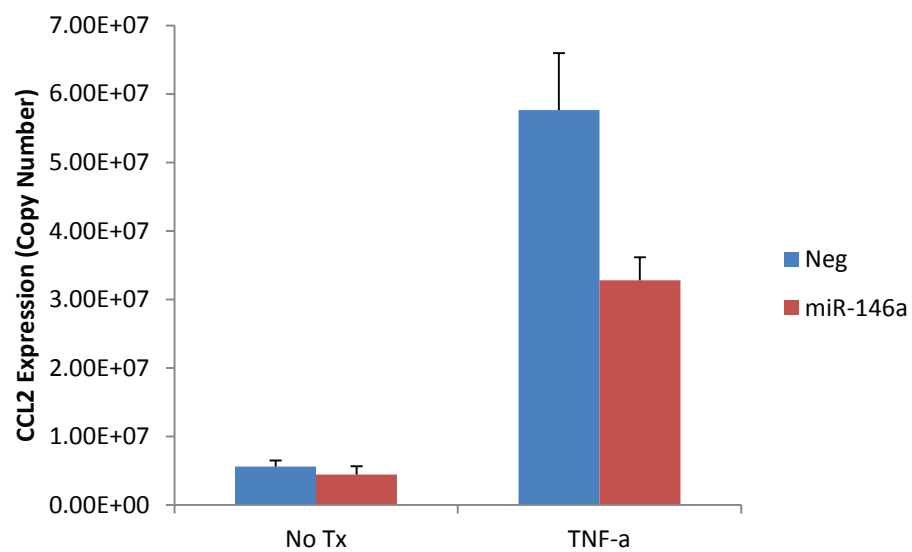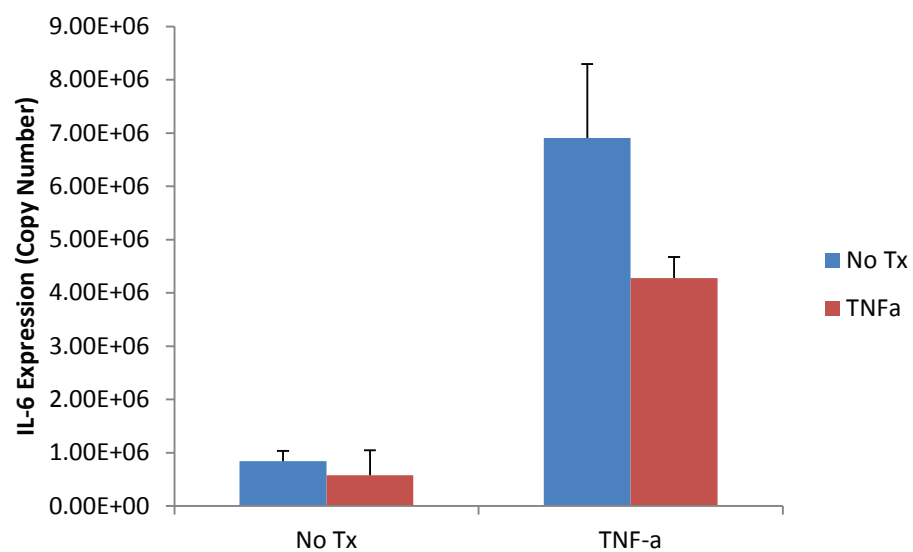

Supplement: S3 Fig — (PDF) [file pone.0205434.s003.pdf]
